# RETRACTION: Prevalence of Surgical Site Infection and Risk Factors in Patients after Knee Surgery: A Systematic Review and Meta‐Analysis

**DOI:** 10.1111/iwj.70269

**Published:** 2025-03-03

## Abstract

**RETRACTION**: MahdiabadiM. Z. , FarhadiB. , ShahroudiP. , MohammadiM. , OmraniA. , MohammadiM. , PourN. H. , HojjatiH. , NajafiM. , TeimooriZ. M. , FarzanR. , and SalehiR. , “Prevalence of Surgical Site Infection and Risk Factors in Patients after Knee Surgery: A Systematic Review and Meta‐Analysis,” International Wound Journal 21, no. 2 (2024): e14765, 10.1111/iwj.14765.38351472 PMC10864688

The above article, published online on 13 February 2024, in Wiley Online Library (http://onlinelibrary.wiley.com/), has been retracted by agreement between the journal Editor in Chief, Professor Keith Harding; and John Wiley & Sons Ltd. A third party reported to the journal that they had found evidence of excessive self‐citations in the reference list of this article. The publisher did not confirm the evidence of excessive self‐citations. Upon further investigation, the publisher also concluded that this article was accepted solely on the basis of a compromised peer review process. In addition, the investigation found significant textual overlap between the methods and discussions sections of this article and another article by many of the same authors (Cheng et al. 2024 [https://doi.org/10.1111/iwj.14350]). In view of the clear evidence of compromised peer review, the parties agreed that the paper must be retracted. The authors disagree with the retraction.

**RETRACTION**: M. Z. Mahdiabadi , B. Farhadi , P. Shahroudi , M. Mohammadi , A. Omrani , M. Mohammadi , N. H. Pour , H. Hojjati , M. Najafi , Z. M. Teimoori , R. Farzan , and R. Salehi , “Prevalence of Surgical Site Infection and Risk Factors in Patients after Knee Surgery: A Systematic Review and Meta‐Analysis,” International Wound Journal 21, no. 2 (2024): e14765, 10.1111/iwj.14765.38351472 PMC10864688

The above article, published online on 13 February 2024, in Wiley Online Library (http://onlinelibrary.wiley.com/), has been retracted by agreement between the journal Editor in Chief, Professor Keith Harding; and John Wiley & Sons Ltd. A third party reported to the journal that they had found evidence of excessive self‐citations in the reference list of this article. The publisher did not confirm the evidence of excessive self‐citations. Upon further investigation, the publisher also concluded that this article was accepted solely on the basis of a compromised peer review process. In addition, the investigation found significant textual overlap between the methods and discussions sections of this article and another article by many of the same authors (Cheng et al. 2024 [https://doi.org/10.1111/iwj.14350]). In view of the clear evidence of compromised peer review, the parties agreed that the paper must be retracted. The authors disagree with the retraction.

